# Human olfactory discrimination of genetic variation within *Cannabis* strains

**DOI:** 10.3389/fpsyg.2022.942694

**Published:** 2022-10-28

**Authors:** Anna L. Schwabe, Samantha K. Naibauer, Mitchell E. McGlaughlin, Avery N. Gilbert

**Affiliations:** ^1^School of Biological Sciences, University of Northern Colorado, Greeley, CO, United States; ^2^Headspace Sensory LLC, Fort Collins, CO, United States

**Keywords:** *Cannabis*, genetics, aroma, olfaction, organoleptic, properties, genotype

## Abstract

*Cannabis sativa* L. is grown and marketed under a large number of named strains. Strains are often associated with phenotypic traits of interest to consumers, such as aroma and cannabinoid content. Yet genetic inconsistencies have been noted within named strains. We asked whether genetically inconsistent samples of a commercial strain also display inconsistent aroma profiles. We genotyped 32 samples using variable microsatellite regions to determine a consensus strain genotype and identify genetic outliers (if any) for four strains. Results were used to select 15 samples for olfactory testing. A genetic outlier sample was available for all but one strain. Aroma profiles were obtained by 55 sniff panelists using quantitative sensory evaluation of 40 odor descriptors. Within a strain, aroma descriptor frequencies for the genetic outlier were frequently at odds with those of the consensus samples. It appears that within-strain genetic differences are associated with differences in aroma profile. Because these differences were perceptible to untrained panelists, they may also be noticed by retail consumers. Our results could help the cannabis industry achieve better control of product consistency.

## Introduction

*Cannabis sativa* L. has been cultivated for thousands of years as a seed and fiber crop as well as for the psychoactive effects of Δ9-tetrahydrocannabinol (THC; Clarke and Merlin, 2013). The number of named cultivars (commonly referred to as strains) is enormous: At least 600 have been described in the scientific literature (Rahn et al., 2016), while an online database lists over 2,900 (Wikileaf, 2022). Hybrid crosses have resulted in strains that vary in growth form as well as phenotypic traits such as cannabinoid and terpene content (Elzinga et al., 2015; Fischedick, 2017; Jikomes and Zoorob, 2018; Orser et al., 2018).

Strain names are the basis of retail product identification in jurisdictions where *Cannabis* is legal (Gilbert, 2020). As such they are associated with user-relevant attributes such as scent, flavor, appearance, and psychoactive effect. Yet strain names cannot be trademarked under current U.S. law and can be assigned capriciously by breeders, growers, and retailers. Nevertheless, consumers reasonably expect products sold under a given name to have consistent attributes. Ensuring consistency and quality in a psychoactive product is in the interests of consumers and the industry. Information regarding the extent of genetic and sensory variation between and within marketed strains could help consumers make more informed purchasing decisions and cultivators achieve more consistent crops. Our study addresses the physical basis of consistency in one element of *Cannabis* product quality, namely aroma.

Recent studies (Sawler et al., 2015; Lynch et al., 2016; Soler et al., 2017; Schwabe and McGlaughlin, 2019) have found genetic differences within named *Cannabis* strains, an unexpected result given that commercial growers predominantly use clonal propagation in order to produce more uniform product (Campbell et al., 2019). The existence of within-strain genetic differences raises the possibility that corresponding phenotypic variation—perhaps including measurable alterations in aroma—may be present as well. Verifying the presence and extent of such variation is central to establishing quality attributes for commercial *Cannabis* (Sarma et al., 2020).

Scent has a long history of use as a taxonomic marker in plants. Darwin (1876) drew attention to correlations between floral scent and color, and the sensory abilities of insect pollinators. Gilbert (1932) offered a classification of the smells associated with various mushrooms, and Heim (1957, Tome I, pp. 141-150) noted that species of the European mushroom genus *Entholoma* each have a distinctive aroma. In their landmark volume on odor description and classification, Harper et al. (1968) reviewed floral aroma as a case of special interest. In *Cannabis*, the link between scent and strain has become more salient as the industry attempts to broaden the quality focus to attributes other than THC content (Oswald et al., 2021).

While smell is an inherently subjective experience, the science of psychophysics allows odor perceptions to be quantified under standardized experimental conditions. Quantitative sensory analysis reveals consistent results above and beyond individual variation in response. This approach recently has been used to characterize the aroma of dried flower across *Cannabis* strains. Gilbert and DiVerdi (2018) reported two major aroma profiles: one described as earthy, woody, and herbal (Cluster A), and the other described as citrus, lemon, sweet, and pungent (Cluster B). They studied single samples of 11 strains, along with two samples of one strain (“Durban Poison”) purchased from different growers/retailers. Both samples of “Durban Poison” were statistically grouped in Cluster B. New samples of two strains were re-examined in a subsequent study (Gilbert and DiVerdi, 2019), with the new samples producing the same aroma profiles as before. These results suggest a certain degree of aroma consistency within named strains. However, the extent and source of this variation remains an open question.

Several studies have investigated *Cannabis* chemotypes (the chemical profile of a strain), often with a focus on terpenes, the volatile compounds that are important in creating distinctive strain-specific aromas. Variation in terpene profiles has been documented between strains described as “indica” or “sativa” (Watts et al., 2021), in *Cannabis* groups defined by the relative abundance of THC and CBD (Mudge et al., 2019; Smith et al., 2022), among different regions of the United States (Smith et al., 2022), and in the effects reported by medical *Cannabis* patients (Lewis et al., 2018). Mudge et al. (2019) proposed that the diversity of *Cannabis* aromas observed today is a product of selection for scents believed to be associated with specific THC levels.

Trends in plant breeding may be driven in part by the role of aroma in consumer purchasing decisions (Boehnke et al., 2019; de la Fuente et al., 2020) as well as by consumer beliefs linking specific strain aroma profiles to expectations regarding psychoactive potency (Gilbert and DiVerdi, 2018). Meanwhile the industry has begun to emphasize product quality measures other than THC, with one popular website allowing users to sort strains by aroma descriptors (Weedmaps, 2022) that are nearly identical to those validated in the scientific literature (Gilbert and DiVerdi, 2018, 2019). Given the economic importance of aroma and strain identity, we wondered if a genetically anomalous sample of a given strain would smell different from samples that were genetically cohesive. To address this question experimentally, molecular genotyping and olfactory phenotyping techniques were combined in a two-part study. In Study 1, 32 samples from four candidate strains of *Cannabis* were genotyped using 10 variable microsatellite markers (Schwabe and McGlaughlin, 2019) in order to identify a consensus genotype and genetic outliers for olfactory evaluation. In Study 2, 15 samples from the four strains were evaluated by sensory judges, who characterized the aroma using a standardized ballot. The results were used to compare the aroma profiles of genetic consensus and genetic outlier samples within each strain.

## Study 1: Genetic assessment

For this study, we gathered multiple samples of four *Cannabis* strains from different sources in Colorado to be analyzed for genetic similarity using 10 variable microsatellite makers. The results were used to establish a set of consensus samples and (where possible) a genetic outlier for each strain.

### Methods

#### Strain selection

Strain availability (Weedmaps, 2022) and scent descriptions (Leafly, 2019) were researched online and cross-referenced with published data (Gilbert and DiVerdi, 2018, 2019) in order to arrive at four candidate strains (Table 1). “Durban Poison” was selected to represent the citrus, lemon, sweet, and pungent group (Cluster B), while “OG Kush” and “Mob Boss” were selected to represent the earthy, woody, and herbal group (Cluster A; Gilbert and DiVerdi, 2018). The aroma profile of “Blue Dream” had not previously been analyzed, however the online odor descriptors of berry, blueberry, and sweet (Leafly, 2019) indicated this strain might have a scent unlike the other candidates.

**Table 1:** Candidate strains, number of samples in each, their scent cluster identification, and odor description (from Gilbert and DiVerdi, 2018, 2019).

Strain	Samples (*n*)	Aroma cluster	Descriptors
OG Kush	8	A	earthy, woody, herbal
Mob Boss	6	A	earthy, woody, herbal
Durban Poison	8	B	citrus, lemon, sweet, pungent
Blue Dream	10	(not yet tested)	(not available)

#### Sample acquisition

A total of 32 retail *Cannabis* samples were purchased from 21 state-licensed recreational dispensaries in six Colorado cities (Appendix 1). Dispensaries were chosen based solely on the availability of strains. A minimum of six samples of each strain were collected. Each sample weighed 2 grams and was labeled “SN” to indicate eligibility for the olfactory evaluation. Additional samples (labeled “GN”) were included to assess within-strain genetic variability, but were not used in the olfactory portion of this study.

#### DNA extraction and analysis

DNA was extracted using a modified CTAB extraction protocol (Doyle and Doyle, 1987) with 0.035–0.100 grams of dried flower tissue per extraction. Ten primers developed *de novo* from the “Purple Kush” genome were used to amplify DNA fragments containing variable microsatellite regions.

### Results

#### Genetic relatedness

GENELEX ver. 6.4.1 (Peakall and Smouse, 2006; Peakall and Smouse, 2012) was used to calculate Lynch and Ritland (1999) pairwise genetic relatedness value (r) of all samples within a strain. A value of r = 1.00 indicates identical individuals, as observed in clones; r < 0 indicates a very low level of relatedness. “Durban Poison” samples 1SN, 4SN and 5SN were identical (r = 1.00). “OG Kush” samples 2SN and 3SN, and samples 6SN and 8GN, had a high level of genetic relatedness (r = 1.00 and r = 0.91, respectively); pairwise relatedness between the remaining samples was low to moderate (0.06 ≤ r ≤ 0.75). “Blue Dream” samples 1SN, 2SN, 4SN, 5SN, 6SN, and 8GN were genetically identical (r = 1.00); sample 3SN had very low relatedness to that group of samples (r = − 0.21). “Mob Boss” samples 1SN and 3SN were genetically identical (r = 1.00); sample 5SN had very low relatedness to each of them (r = −0.22 and r = − 0.17 respectively).

#### Sample clustering based on genetic analysis

Principal Coordinates Analysis was conducted in GENALEX and plotted using the ggplot package in RStudio (Team R, 2015; Wickham, 2016) with 95% confidence interval ellipses around the resulting four groups (Supplementary Figure 1).

#### Sample selection for sensory analysis

Analysis of genetic relatedness (r) and PCoA clearly identified consensus samples and genetic outliers within each strain. The outlier sample of “Durban Poison” (DuPo_8GN) was too small to be included in the olfactory evaluations; however three consensus samples of “Durban Poison” were included in order to address scent variation among identical genotypes. The complete set of 15 samples selected for the sensory study is shown in Table 2.

**Table 2:** The 15 samples selected for olfactory evaluation in Study 2.

Strain	Sample ID
Durban Poison	DuPo_4SN DuPo_4SN DuPo_5SN
OG Kush	OGKu_1SN* OGKu_2SN OGKu_3SN OGKu_4SN
Blue Dream	BlDr_1SN BlDr_3SN* BlDr_4SN BlDr_5SN BlDr_6SN
Mob Boss	MoBo_1SN MoBo_3SN MoBo_5SN*

Asterisk identifies a genetic outlier.

**Figure 1:**
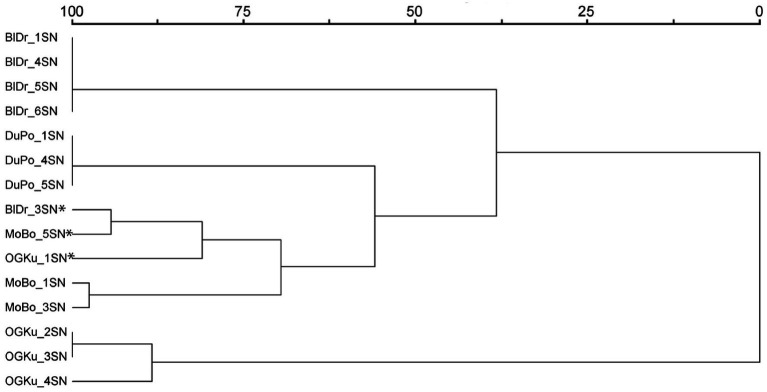
Hierarchical cluster analysis based on genetic similarity. Included are the 15 *Cannabis* samples selected for olfactory evaluation in Study 2. Asterisks identify the genetic outliers. Abbreviations: BlDr = “Blue Dream,” MoBo = “Mob Boss,” OGKu = “OG Kush,” and DuPo = “Durban Poison.”

The 15 samples selected for olfactory evaluation were included in a hierarchical cluster analysis in PC-ORD (McCune and Mefford, 1999) using Ward’s method and Euclidean distance parameters based on pairwise genetic distances output from GENALEX. The resulting dendrogram showed four strain-specific clusters and one mixed-strain cluster (Figure 1). The mixed strain cluster consisted of the three genetic outlier samples.

## Study 2: Olfactory assessment

Having identified an appropriate set of samples in Study 1, we presented them as odor stimuli in a quantitative olfactory evaluation session. Odor judges rated each sample, and the results were used to establish aroma profiles. Our objective was to determine whether the profiles of the genetic outlier samples differed from those of same-strain consensus samples.

### Methods

#### Ethics statement

This study protocol was approved by the Western Institutional Review Board (Puyallup, Washington; WIRB Protocol #20170080). All participants provided informed written consent using a form approved by WIRB. At no time did participants come into direct contact with the *Cannabis* samples.

#### Participants

Participants were recruited from Fort Collins and vicinity. Participants from a previous study (Gilbert and DiVerdi, 2018) who indicated a willingness to participate in further research were re-contacted. A notice (text approved by the institutional review board) was posted to a local online bulletin board. Printed text emphasized “current, former, and non-users all welcome” and that only sniffing was required (“no touching, no smoking, no eating”). All participants were at least 21 years of age, residents of Colorado, and had a self-reported normal sense of smell. Exclusion criteria included self-reported pregnancy, active nasal allergy, current head cold, and age > 50 years. Subjects were paid $20.00 for their participation.

Recruitment criteria for this study were similar to those used in previous work on *Cannabis* aroma (Gilbert and DiVerdi, 2018, 2019, 2020) and are based on those used in routine consumer sensory evaluation to ensure, short of formal clinical assessment of each prospective panelist, that results are not skewed by permanent or temporary smell impairment including age-related decline.

Participants were asked whether they had (a) purchased and (b) smoked or vaped *Cannabis* flower since recreational use became legal in Colorado on January 1, 2014. More extensive (and potentially invasive) queries regarding panelists’ usage history were not made as the association between *Cannabis* use and olfactory abilities is inconclusive (Lötsch and Hummel, 2015; Walter et al., 2017; Tarragon and Moreno, 2019).

#### Odor stimuli

Odor stimuli consisted of 15 *Cannabis* samples drawn from four strains (Table 2). Each stimulus (1 g of dried *Cannabis* flower) was presented in a wide mouth 4 oz. (118 ml) amber glass bottle labeled with a three-digit code. Samples were kept in a freezer at −2° C and thawed at room temperature for two hours before testing. Samples were stored frozen to reduce heat-induced evaporation and oxidation of the volatile components that are the basis of *Cannabis* aroma. Benefits of low temperature storage have been demonstrated for moisture content and cannabinoid concentrations and likely apply to terpenes and terpenoids as well (Das et al., 2022; Meija et al., 2022). The stimuli were exchanged for fresh samples midway through the study.

#### Procedure

Sensory testing was conducted double-blind: samples were assigned a random 3-digit identification code to disguise strain identities from both the test administrator and test participants. Participants rated each sample using a check-all-that-apply (CATA) ballot with 40 previously validated odor descriptors (Gilbert and DiVerdi, 2019; Table 3), presented in alphabetical order on a single screen of a touch-screen device (Apple iPad 2). Sample presentation sequence was randomized for each participant. Data were automatically entered into a spreadsheet; scale presentation and data collection were designed using free online services (Google Forms and Google Sheets).

**Table 3:** Odor descriptors used by sensory panelists to characterize the samples.

Ammonia	Diesel	Mango	Rose
Apricot	Earthy	Menthol	Sage
Berry	Flowery	Mint	Skunk
Blue cheese	Grape	Nutty	Spicy
Butter	Grapefruit	Orange	Sweet
Cheese	Herbal	Peach	Tea
Chemical	Honey	Pepper	Tobacco
Chestnut	Lavender	Pine	Tropical fruit
Citrus	Lemon	Pineapple	Violet
Coffee	Lime	Pungent	Woody

#### Analysis

The sensory data for each strain consists of frequency counts across the 40 odor descriptors. To explore similarities and differences in aroma profile across samples, these counts were analyzed with the Hierarchical Cluster procedure in IBM SPSS Version 24, using between-groups linkage on squared Euclidean distances.

### Results

#### Subject demographics

Fifty-five people (33 men, 22 women; mean age 29.5 ± 7.8 years) were tested. All but eight had purchased *Cannabis* since recreational use was legalized in Colorado on January 1, 2014, and all but five had smoked it. The high rates of purchase (85.5%) and use (90.9%) occurred despite efforts to recruit former and non-users as well. Seven subjects (12.7%) had taken part in previous studies (Gilbert and DiVerdi, 2018, 2019). The sample size of the present study (n = 55) is in keeping with the entirety of the *Cannabis* sensory evaluation literature: 62 (Gilbert and DiVerdi, 2018), 52 (Gilbert and DiVerdi, 2019), 21 (Gilbert and DiVerdi, 2020), and 10 (Doty et al., 2004).

#### Aroma profiles mapped across strains and samples

**Figure 2:**
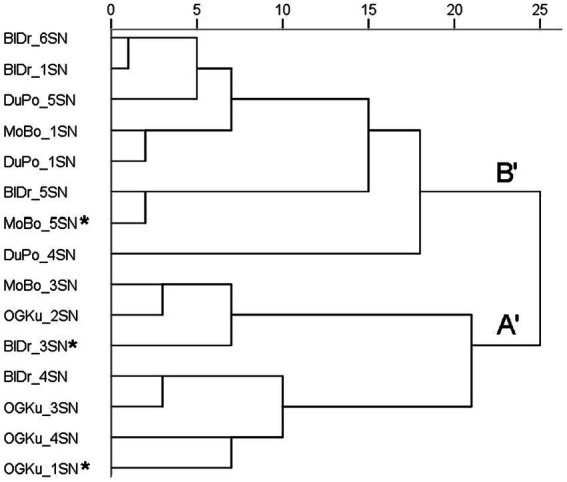
Hierarchical cluster analysis (HCA) of odor descriptor data for the 15 samples in Study 2. Included are the 15 samples in Study 2. The main clusters are labeled A′ and B′. Asterisks indicate genetic outliers; all “Durban Poison” samples were genetically identical. Distances are rescaled to 25.

A sample’s aroma profile can be represented as the frequency counts of its endorsed odor descriptors (Gilbert and DiVerdi, 2018) or the means of rating scale points assigned to each descriptor (Gilbert and DiVerdi, 2019). In previous studies, hierarchical cluster analysis sorted the profiles into two clusters. Strains in Cluster A were described as predominantly earthy, woody, and herbal; those in Cluster B were described as citrus, sweet, pungent, and lemon. Descriptor data in the present study also yielded a dendrogram with two clusters (Figure 2), labeled here A′ and B′. The predominant descriptors for Cluster A′ were earthy, herbal, and woody; those for Cluster B′ were flowery, herbal, sweet, citrus, and earthy. Aroma profiles A and A′ have the same three top-rated descriptors. Profiles B and B′ share sweet and citrus as top-rated descriptors, but B′ also includes flowery, herbal, and earthy. In summary, the major olfactory divisions observed in the present study are consistent with those reported previously.

All four samples of “OG Kush” were located within Cluster A′. This aligns with its previous placement in Cluster A (Gilbert and DiVerdi, 2018). All three samples of “Durban Poison” were located within Cluster B′, aligning with its previous placement in Cluster B (Gilbert and DiVerdi, 2018). “Mob Boss” was previously in Cluster A (Gilbert and DiVerdi, 2018). Here, one consensus sample of “Mob Boss” (MoBo_3SN) was located within Cluster A′; the other consensus sample was assigned to Cluster B′, along with the genetic outlier which received markedly different ratings (see below). This is the first olfactory analysis of “Blue Dream.” Two samples (including the genetic outlier) were assigned to Cluster A′; the other three were assigned to Cluster B′. While all samples were characterized as herbal and earthy, those assigned to Cluster A were also highly rated for either sweet or flowery.

#### Aroma profiles and within strain genetic differences

**Figure 3:**
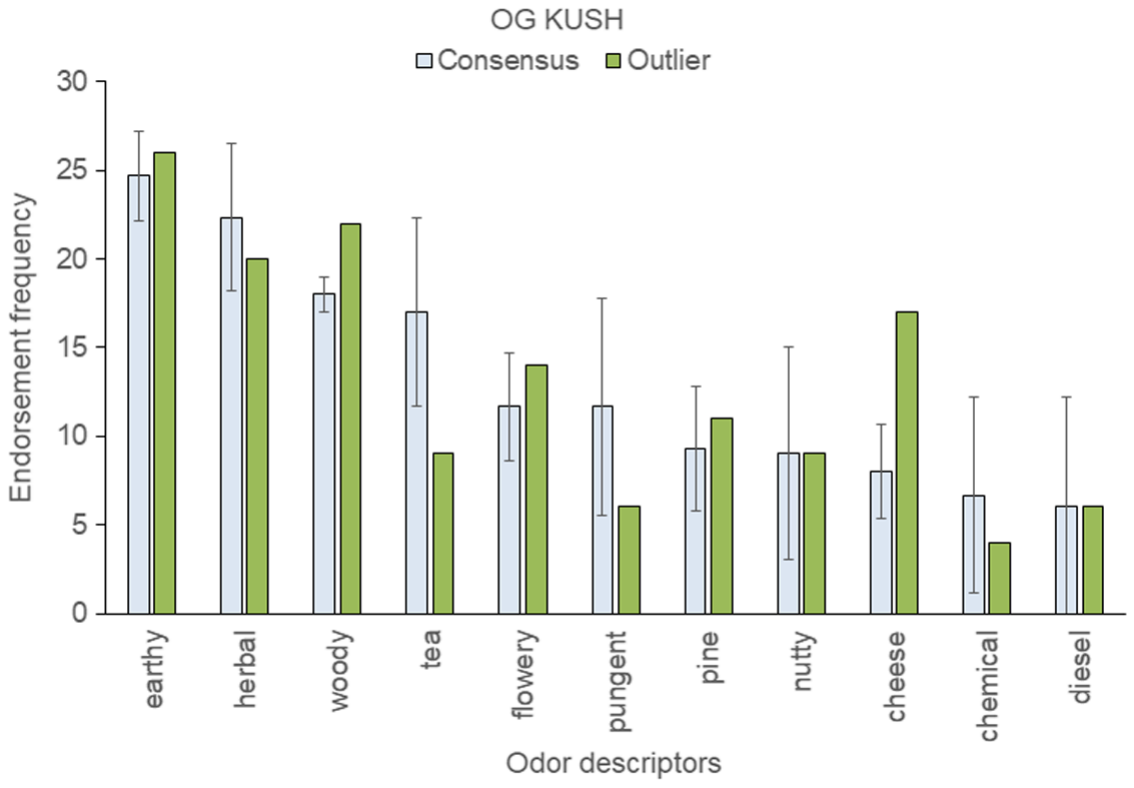
Top-rated odor descriptors for “OG Kush” with error bars indicating standard deviation from the mean of the three consensus samples.

**Figure 4:**
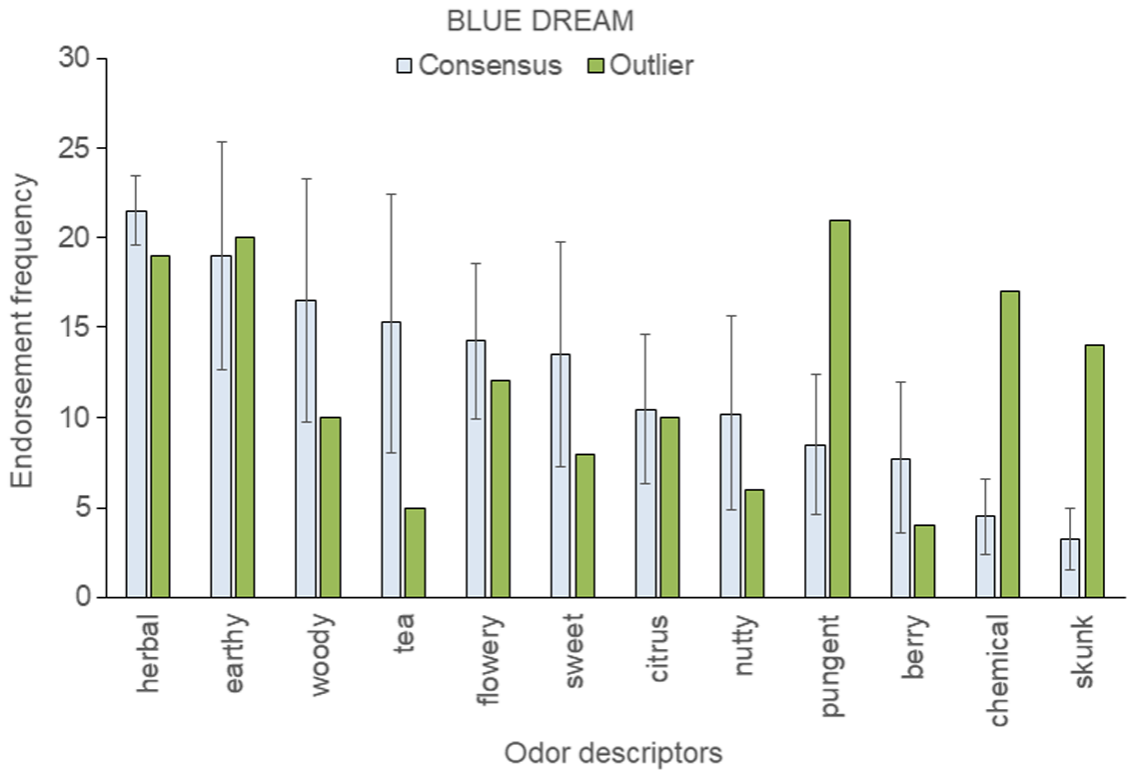
Top-rated odor descriptors for “Blue Dream” with error bars indicating standard deviation from the mean of the four consensus samples.

**Figure 5:**
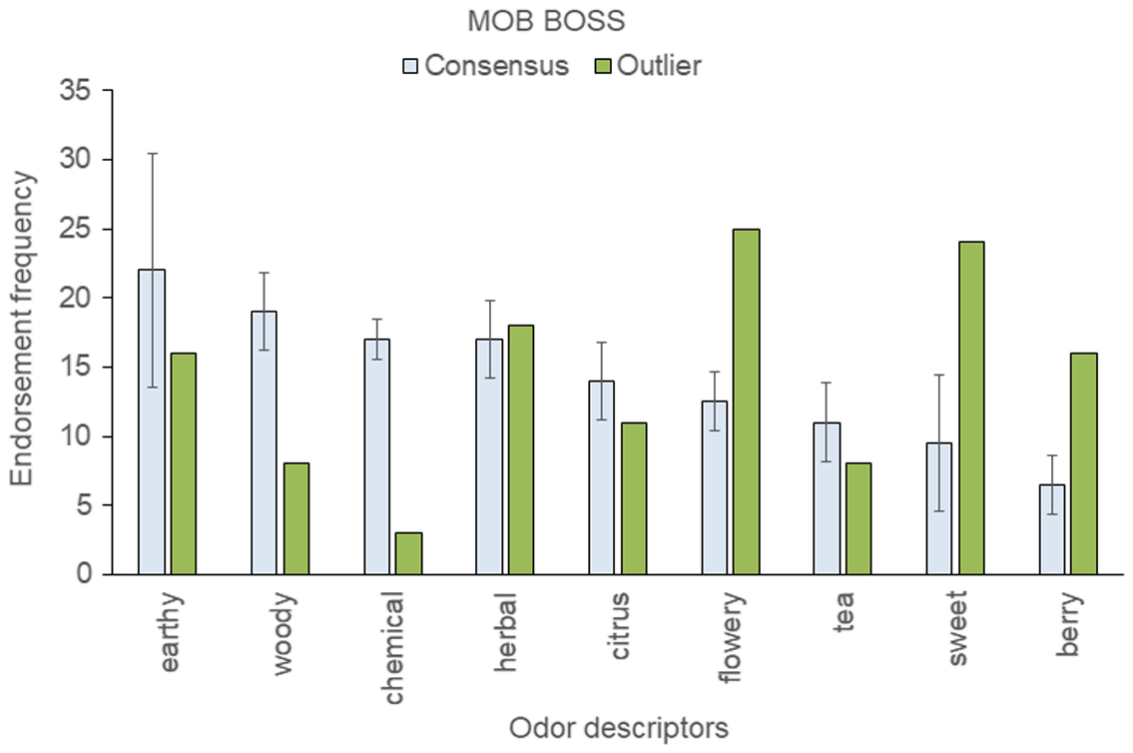
Top-rated odor descriptors for “Mob Boss” with error bars indicating standard deviation from the mean of the two consensus samples.

**Figure 6:**
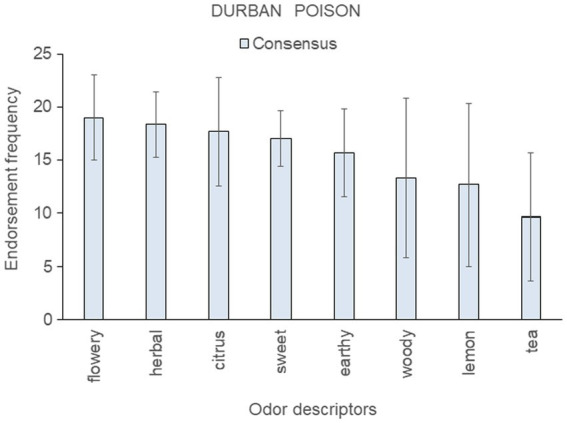
Top-rated odor descriptors for “Durban Poison” with error bars indicating standard deviation from the mean of the three consensus samples; no genetic outlier sample was available for this strain.

To assess within-strain aromatic differences with respect to the genetic outlier, we analyzed the five most frequently endorsed descriptors for each sample; these were then pooled within each strain. This resulted in 12 pooled descriptors for “Blue Dream,” eleven for “OG Kush,” nine for “Mob Boss,” and eight for “Durban Poison.” The mean (± SD) frequency count for each top-rated descriptor was then calculated across a strain’s consensus samples, and compared to the results of the outlier sample (see Figures 3–6).

An outlier sample descriptor was considered anomalous if it fell more than one standard deviation beyond the consensus mean. By this standard, the “OG Kush” outlier smelled more cheesy and woody, and less tea-like, than the consensus samples (Figure 3). The fact that cheese was a frequently endorsed descriptor for the anomalous genetic sample is noteworthy: cheese was not a highly ranked descriptor for any of the 18 strains tested previously (Gilbert and DiVerdi, 2018, 2019).

Five top-ranked descriptors for the “Blue Dream” genetic outlier were outside the standard deviation of the consensus sample mean: the outlier smelled more pungent, chemical, and skunk-like than the four consensus samples, but was less herbal and tea-like (Figure 4). The HCA dendrogram (Figure 2) aligns two “Blue Dream” samples, including the genetic outlier, with Cluster A′, and the other three samples with Cluster B′. Despite “Blue Dream” samples appearing in both the A′ and B′ clusters, the genetic outlier displays unique and marked differences from the mean consensus descriptors.

The “Mob Boss” genetic outlier was strikingly more flowery, sweet, and berry-like, and less woody, chemical, citrus, and tea-like than the consensus samples (Figure 5). The two consensus genetic samples of “Mob Boss” tested here fit the Cluster A profile: earthy, woody, and herbal were top scoring descriptors for each. In contrast, the description of the genetic outlier sample as flowery, sweet, herbal, berry was at odds with both the consensus samples and the previously established Cluster A profile for “Mob Boss.”

Results for the three consensus samples of “Durban Poison” are shown in Figure 6 (no genetic outlier was available for this strain). Citrus, sweet, and lemon were among the top-ranked descriptors for these samples, consistent with the strain belonging to aroma Cluster B (Gilbert and DiVerdi, 2018) and Cluster B′ (this paper, Figure 2). However, these samples also scored highly on herbal, earthy, and woody, descriptors more typical of Cluster A.

#### Discussion

The aroma profiles of “Durban Poison” and “OG Kush” lined up well with results of previous studies; those of “Mob Boss” less so, with genetic consensus samples assigned to both Cluster A′ and B′. This was the first study to evaluate the aroma profile of “Blue Dream.” The results were ambiguous: two samples were located in Cluster A′ and three in Cluster B′. The “Blue Dream” results are particularly interesting given that all consensus samples of “Blue Dream” were genetically identical, but one of four samples was placed in Cluster A′ with the genetic outlier. The lack of consistent aroma profile cluster assignment in genetic consensus samples from two of the four strains examined raises questions about how non-genetic factors (e.g., differences in growth conditions, harvest time, post-harvest processing, etc.) can impact aroma.

By focusing on the top-scoring odor descriptors within each strain, we observed substantial differences in aroma profile between the consensus samples and the genetic outlier. This suggests that variability in the neutral genetic markers we analyzed may be associated with phenotypic variations in aroma production.

## General discussion

We sought to determine whether within-strain genetic variation in *Cannabis* manifests as discrepant aroma production. In Study 1, 10 neutral genetic markers were used to characterize similarities and differences among samples within four strains. The results confirmed previous observations of within-strain genetic variability in samples purchased in the retail market (Schwabe and McGlaughlin, 2019).

The results of Study 1 enabled us to identify genetically cohesive and outlier samples for three strains, along with genetically identical samples for a fourth. In Study 2, these samples were presented as odor stimuli for sensory evaluation by human panelists. The odor results confirmed the high-level organization of *Cannabis* olfactory space observed previously (Gilbert and DiVerdi, 2018, 2019), namely that aroma profiles form two distinct clusters, one described as earthy, woody, and herbal (Cluster A), and the other described as citrus, lemon, sweet, and pungent (Cluster B). Further, we found that two of the named strains tested (“OG Kush” and “Durban Poison”) were assigned to the same clusters as before (Clusters A/A′ and B/B′ respectively), while the results for “Mob Boss” were ambiguous. “Blue Dream,” described here for the first time, appeared in both aroma clusters. While this result could cut against the generality of the Cluster A versus Cluster B distinction, the impact of non-genetic factors (e.g., differences in growth conditions, harvest time, post-harvest processing, etc.) on *Cannabis* flower aroma remains under explored.

Variation in ratings is inherent in sensory evaluation. Some can be attributed to the sensory response of judges (e.g., differences in olfactory discrimination and use of descriptors), and some to non-genetic variation in the physical samples resulting from, for example, differences in terpene and cannabinoid content due to harvest date (Pacifico et al., 2008; Potter, 2014; Aizpurua-Olaizola et al., 2016; Richins et al., 2018; Booth et al., 2020), growth conditions (Potter, 2014; Jin et al., 2019), and post-harvest processing (Jin et al., 2019; Kovalchuk et al., 2019). In the current work, the genetic outlier samples displayed strikingly atypical aroma profiles when compared to the genetic consensus samples suggesting that variation in neutral genetic markers may be associated with differences in the composition, production, or release of odorous volatiles from dried *Cannabis* flower.

Mono- and sesqui-terpenes are the most abundant volatile compounds in *Cannabis* (Rice and Koziel, 2015; Fischedick, 2017; Orser et al., 2018) and are thought to be responsible for the characteristic odor of mature and dried flowers. Strain differences in terpene composition have been observed (Casano et al., 2011; Lewis et al., 2018; Mudge et al., 2019; Smith et al., 2022) and crowd-sourced ratings have been used to sort strains according to sensory similarity (de la Fuente et al., 2020). However, the association between specific terpenes and a strain’s aroma profile remains speculative pending definitive studies using gas chromatography-olfactometry as has been done for the cones of the hop plant (Steinhaus and Schieberle, 2000). In addition, a new family of prenylated volatile sulfur compounds (VSCs) has been discovered in *Cannabis*; these appear to contribute the “skunky,” “diesel,” or “gassy” notes that are conspicuous in some strains (Oswald et al., 2021). Volatile sulfur compounds are present at low concentration, confirming the perfumer’s dictum that chemical abundance is not a measure of odor impact: Less abundant molecules may make outsized contributions to aroma if they have low thresholds for odor perception.

Variation in terpene and VSC production (and thus aroma) also may have several non-genetic sources. These include aspects of growing conditions, such as nutrient supply, light regimen, harvest timing, and post-harvest flower processing (Cervantes, 2006), and even flower position along the stem (Namdar et al., 2018). As is the case for grapes (Jackson and Lombard, 1993; Roujou de Boubée et al., 2000) and hops (Patzak et al., 2010; Pavlovic et al., 2012; Sharp et al., 2014), phytochemical production in *Cannabis* is likely influenced by a host of environmental factors (Figueiredo et al., 2008). The variability observed within genetic consensus samples—as placement in Cluster A′ versus B′ and in judges’ mean scent ratings—lends further support to the role of non-genetic sources on *Cannabis* aroma. Future research should examine how controlled changes in non-genetic factors impact aroma profiles within a single genetic background.

In conclusion, we found evidence that within-strain genetic variation in *Cannabis* is associated with altered aroma perception. Phenotypic variation in odor production merits further attention as it is detectable by non-expert consumers and may impact their judgments of product quality and purchasing decisions. Quantified aroma profiles, along with a metric for expected variation, could provide a useful standard for detecting departures from strain-specific aroma character. Within-strain variability can exist within a larger pattern of between-strain consistency. Determining the relative impact of genetic and environmental factors on aroma production could help the *Cannabis* industry achieve better control of product consistency.

Future research could elucidate if strain aroma variation impacts consumer purchasing decisions, the level at which odor divergence can detect inconsistencies with the on-label strain names, and if deviations from an expected aroma profile reduce what consumers are willing to pay.

## Data availability statement

The original contributions presented in the study are included in the article/Supplementary material, further inquiries regarding the genetic data can be directed to the corresponding author.

## Ethics statement

The studies involving human participants were reviewed and approved by Western Institutional Review Board. The participants provided their written informed consent to participate in this study.

## Author contributions

AS conceived the project, provided some funding, recruited participants, collected samples, conducted DNA extractions, designed, and optimized microsatellite primers, compiled and analyzed data, and drafted manuscript content. SN collected samples, and conducted DNA extractions. MM provided some funding, contributed statistical analysis and manuscript revisions. AG provided some funding, recruited participants, conducted the sensory data collection, analyzed sensory data, contributed statistical analysis and manuscript revisions. All authors contributed to the article and approved the submitted version.

## Funding

Partial funding was provided by Headspace Sensory LLC, the University of Northern Colorado Graduate Student Association, the McGlaughlin Lab at the University of Northern Colorado, and by the first author.

## Conflict of interest

AG is Founder and Managing Member of Headspace Sensory, LLC. This study received funding from Headspace Sensory, LLC. The financial involvement of Headspace Sensory, LLC in this research was the funding for research materials and participant incentive in the sensory portion of the study. The funder has the following involvement in the study: provided some funding, recruited participants, conducted the sensory data collection, analyzed sensory data, contributed statistical analysis and manuscript revisions.

The remaining authors declare that the research was conducted in the absence of any commercial or financial relationships that could be construed as a potential conflict of interest.

## Publisher’s note

All claims expressed in this article are solely those of the authors and do not necessarily represent those of their affiliated organizations, or those of the publisher, the editors and the reviewers. Any product that may be evaluated in this article, or claim that may be made by its manufacturer, is not guaranteed or endorsed by the publisher.

## Supplementary material

SUPPLEMENTARY FIGURE S1 Principal Coordinates Analysis of genetic distance among 32 samples representing 30.9% of the genetic variation on Axis 1, and 19.4% of the variation on Axis 2. Clustering indicates genetic similarity. The ellipses represent 95% confidence intervals for each group (“Blue Dream” = blue, “Durban Poison” = red, “Mob Boss” = green, “OG Kush” = purple). Genetic outliers are those which fell outside the confidence intervals.
