# Supplementary figures and images for: Human olfactory discrimination of genetic variation within Cannabis strains
Source: Front Psychol. 2022 Oct 28;13:942694. doi: 10.3389/fpsyg.2022.942694 (PMC9651054; doi:10.3389/fpsyg.2022.942694)

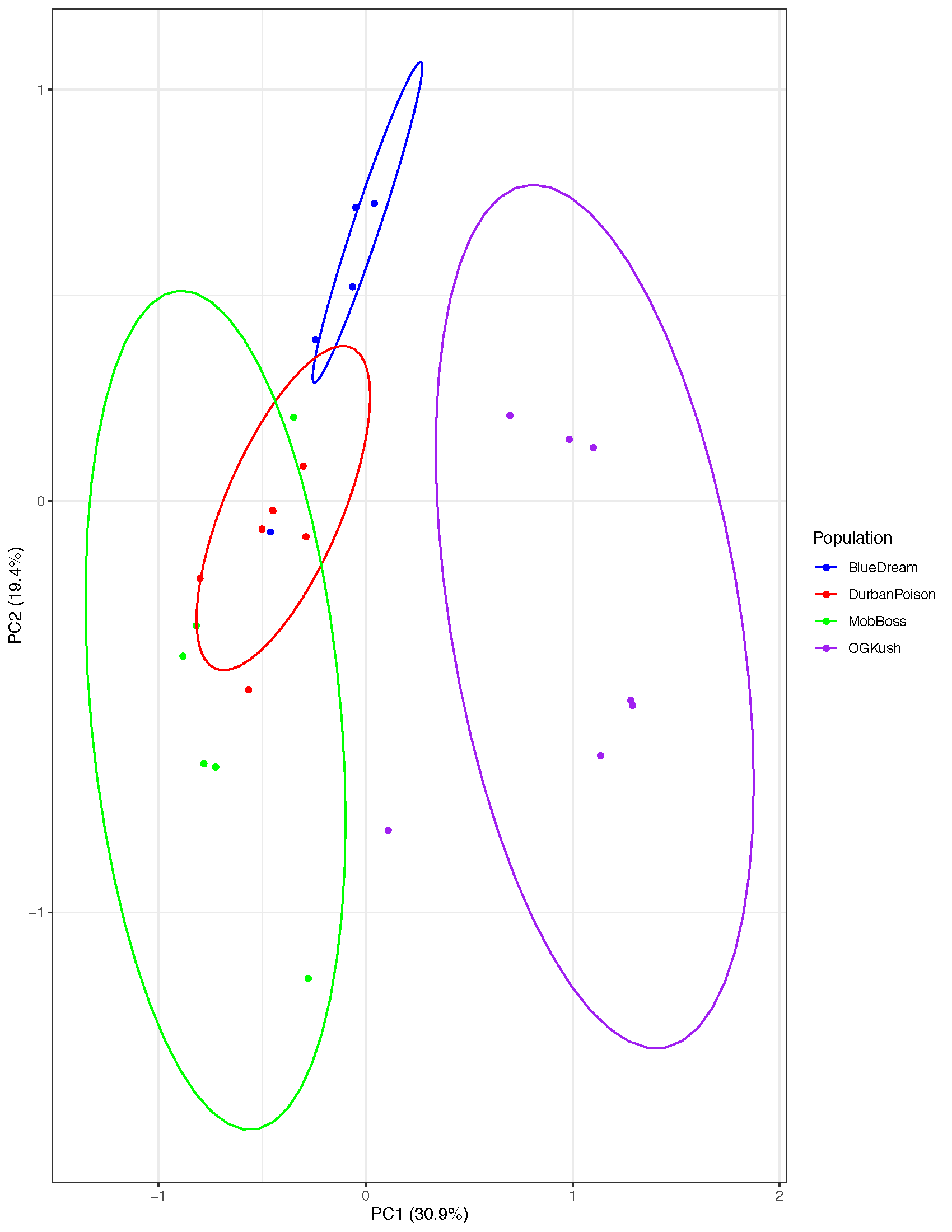

Supplement: SUPPLEMENTARY FIGURE S1 — Principal Coordinates Analysis of genetic distance among 32 samples representing 30.9% of the genetic variation on Axis 1, and 19.4% of the variation on Axis 2. Clustering indicates genetic similarity. The ellipses represent 95% confidence intervals for each group (“Blue Dream” = blue, “Durban Poison” = red, “Mob Boss” = green, “OG Kush” = purple). Genetic outliers are those which fell outside the confidence intervals. [file Image_1.TIFF]
